# Regional variations of lipids in camel milk from Xinjiang, China: a UHPLC–MS/MS-based lipidomics study

**DOI:** 10.3389/fvets.2026.1834007

**Published:** 2026-05-12

**Authors:** Lin Zhu, Di Wen, Zhiwei Li, Xiushan Tan, Yusong Shen, Penglan Dou, Qiaoye Yang, Subinur Kurbanjan, Yanfen Cheng, Changjiang Zang, Fengming Li

**Affiliations:** 1 College of Animal Science, Xinjiang Agricultural University, Ürümqi, China; 2 Xinjiang Branch of National Center for International Research on Animal Gut Nutrition, Xinjiang Agricultural University, Ürümqi, China; 3 Culinary and Catering Management, Xinjiang Vocational University, Ürümqi, China; 4 Laboratory of Gastrointestinal Microbiology, National Center for International Research on Animal Gut Nutrition, Nanjing Agricultural University, Nanjing, China

**Keywords:** Bactrian camel milk, geographical traceability, lipid compounds, lipidomics, oxidized lipids, UHPLC–MS/MS

## Abstract

Camel milk is widely recognized in Xinjiang for its rich nutritional profile and potential health-promoting properties. Here, we integrated lipidomics and oxylipidomics to deconstruct lipid heterogeneity in camel milk from grazing Bactrian camels across three ecologically distinct regions of Xinjiang, China (Yumin, TC; Huocheng, LC; Fuhai, AC). Critically, our sampling strategy encompassed three ecologically distinct counties, spanning over 400 km—a spatial scale sufficient to capture Xinjiang’s biogeographic gradients. We identified 1,286 significantly differential lipids and 71 oxidized lipids, particularly in phospholipids, sphingolipids, and glycerolipids. Eight exploratory candidate lipids were identified for geographical discrimination: PC(37:4COOH), PC(18:0/18:3), DG(19:2/18:1) (AC); PC(16:2/18:3), PE(18:0/22:2CHO), PE(20:0/18:3), DG(16:0/20:0) (TC); PS(18:0/22:4) (LC). Significant differences in polyunsaturated fatty acids (arachidonic acid, docosahexaenoic acid, eicosapentaenoic acid, linoleic acid) and their oxidation products were observed, reflecting regional forage and environmental impacts. Overall, these findings provide a preliminary basis for understanding region-specific lipid variation in camel milk and a descriptive framework for future validation studies on geographical traceability.

## 1 Introduction

According to the China Statistical Yearbook, China had 580,000 Bactrian camels in 2023, with 316,000 in Xinjiang, accounting for approximately 54.48% of the national herd. As the primary distribution region of Bactrian camels in China, Xinjiang’s vast territory and complex geographical environment provide a natural experimental setting for investigating environment-lipid interactions. Camel milk, long recognized by Xinjiang residents for its therapeutic properties, including anti-inflammatory and immunomodulatory effects, serves as a functional food with documented health benefits (1, 2).

Lipids are crucial metabolites that directly reflect metabolic status (3). Geographical region is a multifaceted factor, given significant variations in altitude, climatic conditions, soil types, and other environmental parameters across regions (4).

These environmental gradients directly shape the botanical composition and nutritional quality of regional pastures. Given that camels are free-grazing ruminants, variations in forage intake—particularly in fatty acid profiles and micronutrient content—can significantly alter rumen biohydrogenation pathways and hepatic lipid metabolism, ultimately affecting milk fat synthesis and secretion in mammary epithelial cells (5–7). Consequently, the milk lipidome serves as a direct biochemical record of the camel’s interaction with its specific ecological niche. Unraveling these lipid signatures is therefore essential not only for understanding the camel’s physiological adaptation strategies but also for developing lipid biomarkers for geographical traceability.

Forage intake by camels varies regionally due to distinct vegetation profiles: In Yumin County (TC), semi-desert steppe dominates, featuring drought-tolerant shrubs (e.g., *Artemisia* spp.), semi-shrubs, and grasses (e.g., *Stipa* spp.) that transition between desert and typical steppe. Huocheng County (LC) benefits from the Ili River Valley’s favorable conditions, supporting the highest grassland productivity and significantly higher proportions of high-quality graminoids (e.g., *Stipa*, *Bromus*, *Dactylis*) and legumes. In contrast, Fuhai County (AC) is a typical desert region where super-xerophytic shrubs and semi-shrubs (e.g., *Ceratoides latens*, *Haloxylon ammodendron*, *Anabasis brevifolia*, *Reaumuria soongorica*) are the primary forage and exhibit the lowest grass coverage. Halophytic plants also contribute substantially, with heavy reliance on supplemental feeding during winter. Previous studies have demonstrated that geographical location contributes to compositional variations in human milk (8), bovine milk (9), equine milk (10), Bactrian camel milk (11), and fermented camel milk products (12). These differential lipid compounds may serve as novel biomarkers for distinguishing geographical origins.

Although mass spectrometry-based lipidomics has increasingly been applied to food authentication and geographical origin studies (4, 13), its use in camel milk provenance studies remains scarcely documented. This study aimed to construct regional lipid profiles of camel milk from three distinct regions using integrated untargeted and targeted oxylipidomics. By combining multivariate statistical analyses, we aimed to identify geographically specific differential lipid compounds. Furthermore, metabolic pathway analysis of significantly altered lipids was used to explore region-associated lipid metabolic differences under diverse environmental conditions.

## 2 Materials and methods

### 2.1 Sample collection

Samples were collected from Yumin County (TC), Fuhai County (AC), and Huocheng County (LC) between May 15 and 19, 2024, with collection sites separated by >400 km (Table 1). Ambient temperatures during collection were 16 °C (TC), 17 °C (AC), and 23 °C (LC). TC, at the western margin of the Junggar Basin, is characterized by predominantly calcic Xerosols. AC, on the southern slopes of the Altai Mountains, experiences prolonged, severe winters and supports kastanozems. LC lies within the Ili River Valley, where westerly moisture produces a humid microclimate that supports fertile chernozems.

**Table 1:** Regional characteristics and feeding regimens of camel milk production areas

Xinjiang Uygur autonomous region	Latitude and longitude	Annual precipitation (mm)	Annual temperature (°C)	Altitude (m)	Milking mode	Feeding regimen
TC	E82.884197 N46.170966	280	8.5	730	Hand milking	Grazing
AC	E87.516156 N47.119798	120	3.5	497	Hand milking	Grazing
LC	E80.918881 N44.065351	400	11	665	Hand milking	Grazing

All milk samples were collected during the morning milking session (07:00–09:00) to minimize diurnal variation. Full udder milking was performed for each animal. During the same period, milk samples were collected from Dzungarian Bactrian camels of comparable age, body weight, parity, and days in milk (90–120 days), grazing without supplementation. Eight samples per region (24 total) were collected into 5 mL sterile cryovials, immediately immersed in liquid nitrogen, and stored at −80 °C upon laboratory delivery, pending analysis.

### 2.2 Instruments and reagents

HPLC-grade acetonitrile, methanol, isopropanol, methyl tert-butyl ether (MTBE), formic acid, acetic acid, and ammonium acetate were purchased from Thermo Fisher Scientific (Waltham, MA, USA). Ultrapure water (18.2 MΩ cm) was generated using a Milli-Q system (Millipore, Burlington, MA, USA). The SPLASH™ LIPIDOMIX™ Mass Spec Internal Standard was obtained from Avanti Polar Lipids (Alabaster, AL, USA). Isotope-labeled oxylipid internal standards and the targeted oxylipid analytical service were provided by Novogene (Beijing, China).

### 2.3 Sample extraction

Lipid extraction from milk was adapted from the method of Matyash et al. (14) with modifications. The modifications mainly concerned sample volume, internal standard usage, and solvent volumes adapted for a 100 μL milk aliquot.

#### 2.3.1 Lipid extraction

Aliquots (100 μL) of milk were homogenized with methanol (0.75 mL), MTBE (2.5 mL), and SPLASH™ internal standard (10 μL). After shaking (1 h, 25 °C), phase separation was induced with water (0.625 mL). The upper organic layer was collected after centrifugation (1,000 × *g*, 10 min). The lower phase was re-extracted with 1 mL of the solvent mixture (MTBE/methanol/water, 10:3:2.5 v/v/v), and the upper phase was collected. The combined organic phases were dried under N₂ and reconstituted in isopropanol (100 μL) for LC–MS/MS analysis.

#### 2.3.2 Oxidized lipid extraction

For oxidized lipid analysis, a sequential low-temperature extraction and cleanup procedure was used to enrich free and weakly matrix-associated oxylipins while minimizing artificial oxidation during sample handling. Briefly, 10 μL of a mixed isotope-labeled internal standard solution (1 μg/mL) was added to 100 μL of milk prior to any extraction step to correct for recovery losses and matrix effects. The sample was first diluted with 700 μL of ice-cold 50 mM PBS and centrifuged at 12,000 × *g* for 10 min at 4 °C. In this step, PBS served as a mild aqueous diluent to reduce matrix viscosity and facilitate recovery of the water-compatible oxylipid fraction in the supernatant, rather than as a deliberate precipitation reagent. The remaining pellet was then sequentially extracted with 700 μL of 10% methanol and 700 μL of methanol, with centrifugation after each step under the same conditions to improve recovery of oxylipins associated with proteinaceous and lipid-rich matrix components.

The collected supernatants were combined through sequential loading onto an Atlantis Premier BEH C18 SPE cartridge (1 cc, 30 mg; Waters, USA), preconditioned with 700 μL methanol and equilibrated with 700 μL water. Each fraction was passed through the cartridge twice to improve retention of low-abundance analytes. The residue was additionally washed with 700 μL of 10% methanol, and this wash was also applied to the cartridge. After sample loading, the retained oxylipins were eluted with 700 μL methanol, and the eluate was collected and freeze-dried. The dried residue was reconstituted in water/acetonitrile/acetic acid (60:40:0.02, v/v/v), vortexed thoroughly, and centrifuged at 15,000 rpm for 10 min at 4 °C. The final supernatant was transferred to an autosampler vial for LC–MS/MS analysis. All extraction steps were performed on ice and under dim light to minimize ex vivo oxidation and degradation (15). Accordingly, the measured oxylipid profiles are interpreted here as region-associated differences obtained under controlled analytical conditions rather than as direct evidence of *in vivo* oxidative status.

### 2.4 UHPLC–MS/MS analysis

To ensure data quality, procedural blanks (extraction without a sample) were included in each batch to monitor background contamination. A pooled quality control (QC) sample was prepared by combining equal aliquots of all processed samples. All samples were analyzed in a single randomized injection sequence to minimize instrument drift bias. Two QC injections were acquired at the beginning of the sequence to assess instrument performance and equilibrate the LC–MS system, followed by three additional pooled QC injections used for segmented MS/MS acquisition to support lipid annotation. During routine sample analysis, one pooled QC sample was injected after every three experimental samples to continuously monitor analytical stability.

#### 2.4.1 Untargeted lipidomics

Analysis was performed using ultra-high-performance liquid chromatography–tandem mass spectrometry (UHPLC–MS/MS) (16). UHPLC–MS/MS analyses used a Vanquish UHPLC system with an Orbitrap Q ExactiveTM HF mass spectrometer, using an Accucore C30 column (150 × 2.1 mm, 2.6 μm particle size; Thermo Fisher Scientific, Germany) maintained at 40 °C. The flow rate was 0.35 mL/min, and the injection volume was 5 μL. Mobile phases: buffer A with acetonitrile/water (6/4), 10 mM ammonium acetate, 0.1% formic acid; buffer B with acetonitrile/isopropanol (1/9), 10 mM ammonium acetate, 0.1% formic acid. Gradient: 30% B for 2 min, increasing to 43% B at 5 min, then to 55% B at 5.1 min, 70% B at 11 min, 99% B at 16 min, returning to 30% B at 18.1 min. The Q Exactive™ HF mass spectrometer was operated in both positive and negative ionization modes. The principal source parameters were as follows: sheath gas, 40 arbitrary units; auxiliary gas, 10 arbitrary units in positive mode and 7 arbitrary units in negative mode; spray voltage, ±3.5 kV; capillary temperature, 320 °C; auxiliary gas heater temperature, 350 °C; and S-lens RF level, 50. Full MS scans were acquired over m/z 114–1700 at a resolution of 120,000, with an AGC target of 3e6 and a maximum injection time of 100 ms. Data-dependent MS/MS scans were acquired using normalized collision energies of 22, 24, and 28 eV, an isolation window of 1.0 m/z, an AGC target of 2e5, and a dynamic exclusion time of 6 s.

#### 2.4.2 Targeted oxidized lipidomics

Targeted oxylipid analysis was performed using an ExionLC™ AD system (SCIEX) with a QTRAP® 6,500 + mass spectrometer (SCIEX). Samples were introduced onto a C18 Column (100 × 2.1 mm) maintained at 40 °C with a flow rate of 0.3 mL/min. Mobile phase A consisted of water containing 0.1% formic acid, and mobile phase B consisted of acetonitrile. The gradient program was as follows: 35% B at 0–0.5 min, linearly increased to 95% B at 9.5 min, held at 95% B until 10.5 min, returned to 35% B at 11.0 min, and equilibrated at 35% B until 14.0 min. Oxidized lipids were monitored exclusively in negative ion mode using multiple reaction monitoring (MRM). Source parameters were set as follows: Curtain Gas, 40 psi; Collision Gas, Medium; IonSpray Voltage, −4,500 V; source temperature, 500 °C; and Ion Source Gas 1 and Gas 2, 55 psi. The complete MRM transition list, including analyte name, Q1, Q3, retention time, is provided in Supplementary Table S1.

### 2.5 Lipid identification and quantification

#### 2.5.1 Untargeted lipid identification

Untargeted lipid data were processed using LipidSearch 4.2 (Thermo Fisher Scientific) for peak detection, alignment, annotation, and semi-quantitative analysis. The main processing parameters included precursor and product ion mass tolerances of 5 ppm and a retention time tolerance of 0.05 min. Lipid identifications assigned LipidSearch grades A, B, or C were initially retained when supported by MS/MS information, aligned across samples, and not attributable to background ions detected in blank samples. Because the present study was designed as an exploratory global lipid profiling analysis, grades A–C were used for the initial screening step; however, the candidate discriminatory lipids highlighted in the manuscript were further subjected to manual review and filtering before interpretation. Peak areas were normalized to the most appropriate internal standard in the SPLASH™ LIPIDOMIX™ mixture, and the resulting values were interpreted as internal-standard-normalized semi-quantitative abundances. Because ionization behavior and adduct formation differ among lipid classes and ionization modes, these data were interpreted primarily for within-species or closely related-species comparisons rather than for direct quantitative comparison across different subclasses or across ionization modes. These values are suitable for comparing the same lipid species or closely related species across samples, but direct absolute comparisons across chemically distinct lipid subclasses should be interpreted with caution because of differences in ionization efficiency and standard coverage.

#### 2.5.2 Targeted oxidized lipid identification

Oxylipid data were processed in SCIEX OS 1.4 using a validated MRM library. Chromatographic peaks were integrated and screened using a minimum peak height of 500, a signal-to-noise ratio of 5, and a smoothing point of 1. Relative concentrations of targeted oxylipids were obtained by normalization to the isotope-labeled internal standards included in the assay. Lipid subclasses without class-matched absolute standards, including CL species, were retained only within the exploratory untargeted semi-quantitative framework and were not used to support absolute cross-subclass quantitative conclusions.

### 2.6 Statistical analysis

Raw mass spectrometry data were processed for qualitative and semi-quantitative analysis as described above. Multivariate analyses, including principal component analysis (PCA) and orthogonal partial least-squares discriminant analysis (OPLS-DA), were performed using metaX. Differential metabolites were identified using a t-test (*p-*value) and the following criteria: VIP > 1, *p* < 0.05, and fold change ≥ 2 or ≤ 0.5. Differential oxidized lipids were screened using *p* < 0.05 and fold change ≥ 1.5 or ≤ 0.667. Heatmaps were generated in R using z-scores with Pheatmap, and Pearson correlation was used to assess metabolite relationships, visualized with corrplot, with significance at *p* < 0.05. Lipid annotation used KEGG^1^, HMDB^2^, and LIPID MAPS^3^ for robust lipid identification.

## 3 Results

### 3.1 Validation of analytical methods

**Figure 1:**
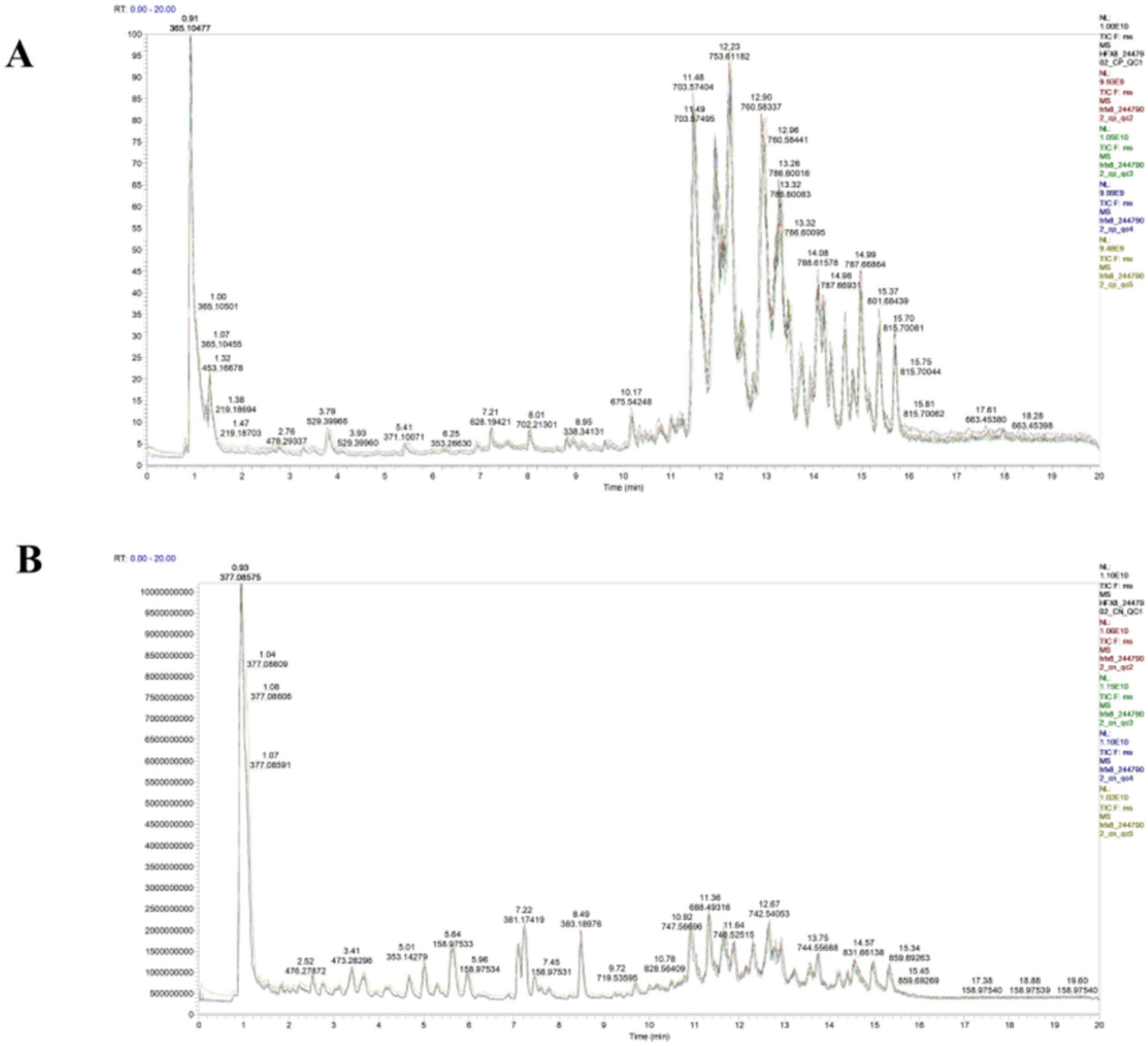
Total ion chromatograms of QC samples in positive ion mode **(A)** and negative ion mode **(B)**.

**Figure 2:**
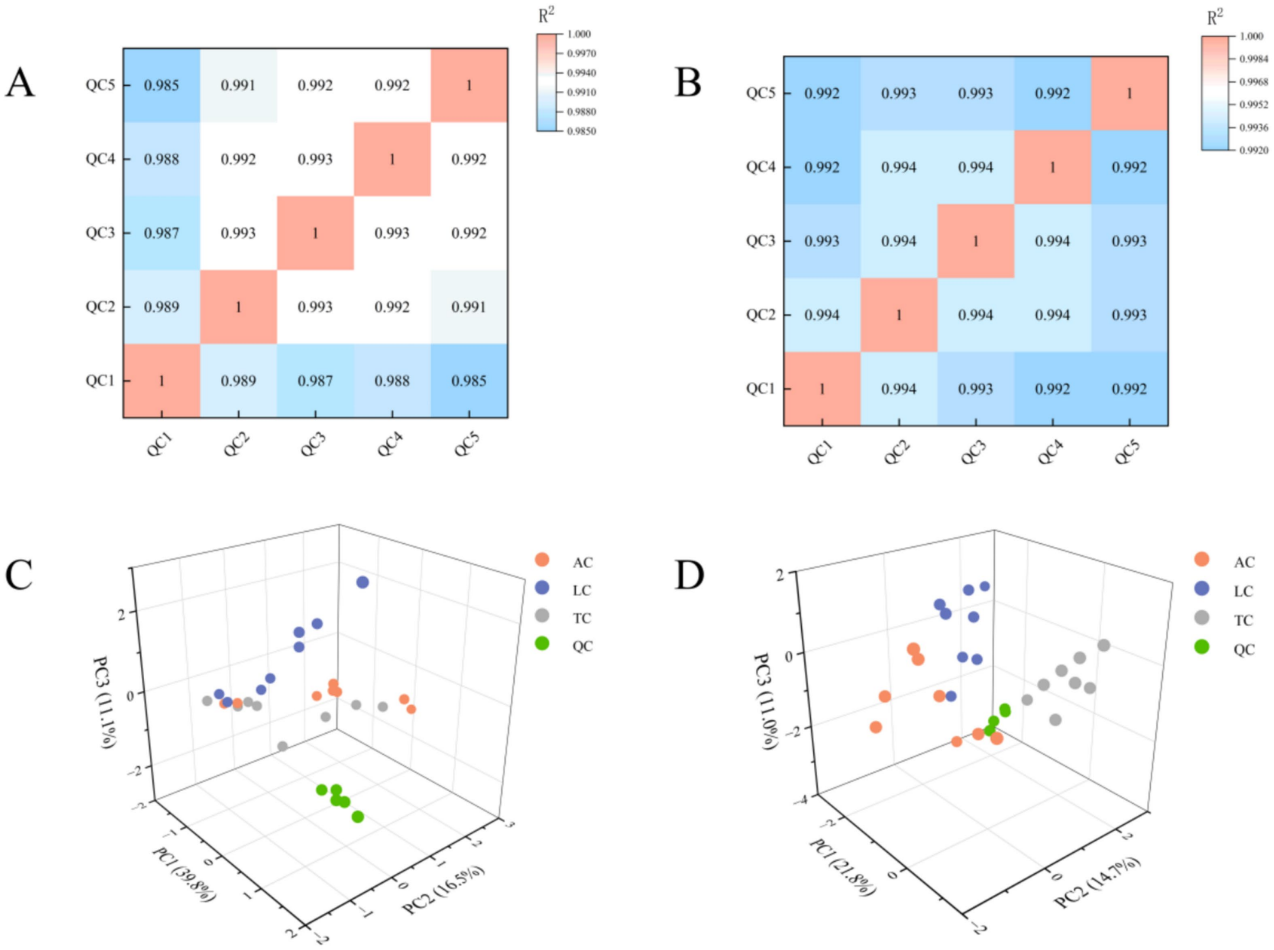
Reliability of the analytical methods. Heat map and 3D PCA score plots of camel milk from different regions under positive ion mode **(A,C)** and negative ion mode **(B,D)**.

**Figure 3:**
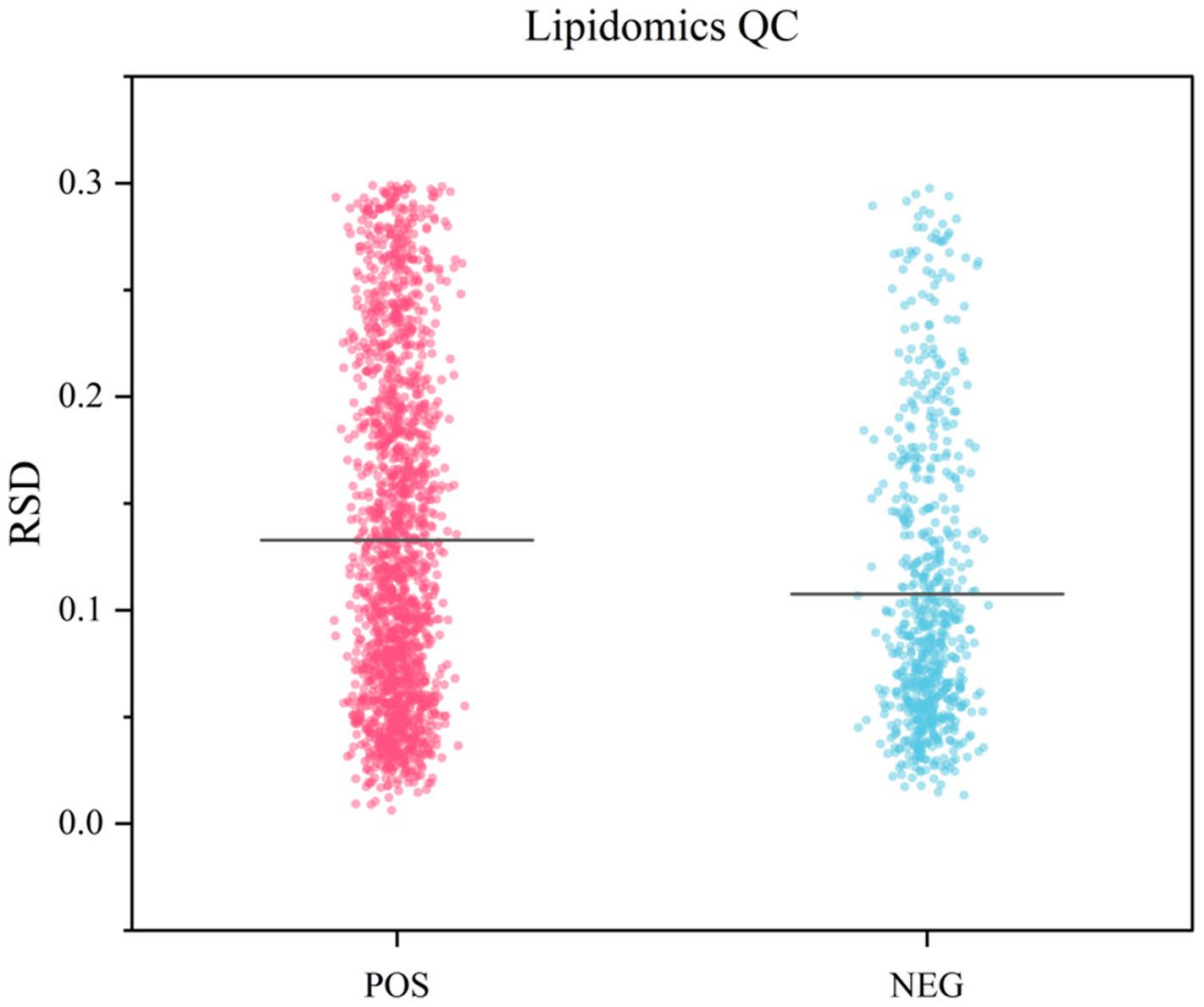
The RSD values of the peak intensities in QC samples.

To evaluate the repeatability of sample preparation and the reliability of analytical methods, we investigated the metabolic profiles of lipid extracts from camel milk across three regions and QC samples. Twenty-four camel milk lipid extracts were pooled into five aliquots and analyzed repeatedly to monitor system stability. As shown in Figure 1, QC samples exhibited substantial overlap in peak area and retention time within total ion chromatograms (TIC), indicating excellent instrument stability. Method reproducibility was assessed through Pearson correlation analysis. Results (Figures 2A,B) showed high within-group correlation coefficients (*R*^2^ > 0.98), indicating a stable process and high data quality. PCA analysis on all samples (Figures 2C,D) revealed tight clustering of QCs within 95% confidence ellipses, validating the robustness of the UHPLC–MS/MS platform. These quality control measures ensured reliable and reproducible lipidomic profiling results. More than 80% of detected lipids showed RSD < 20% in QC samples (Figure 3). This confirms the reproducibility and stability of the analytical platform.

### 3.2 Lipid analysis of camel milk samples from different regions

**Figure 4:**
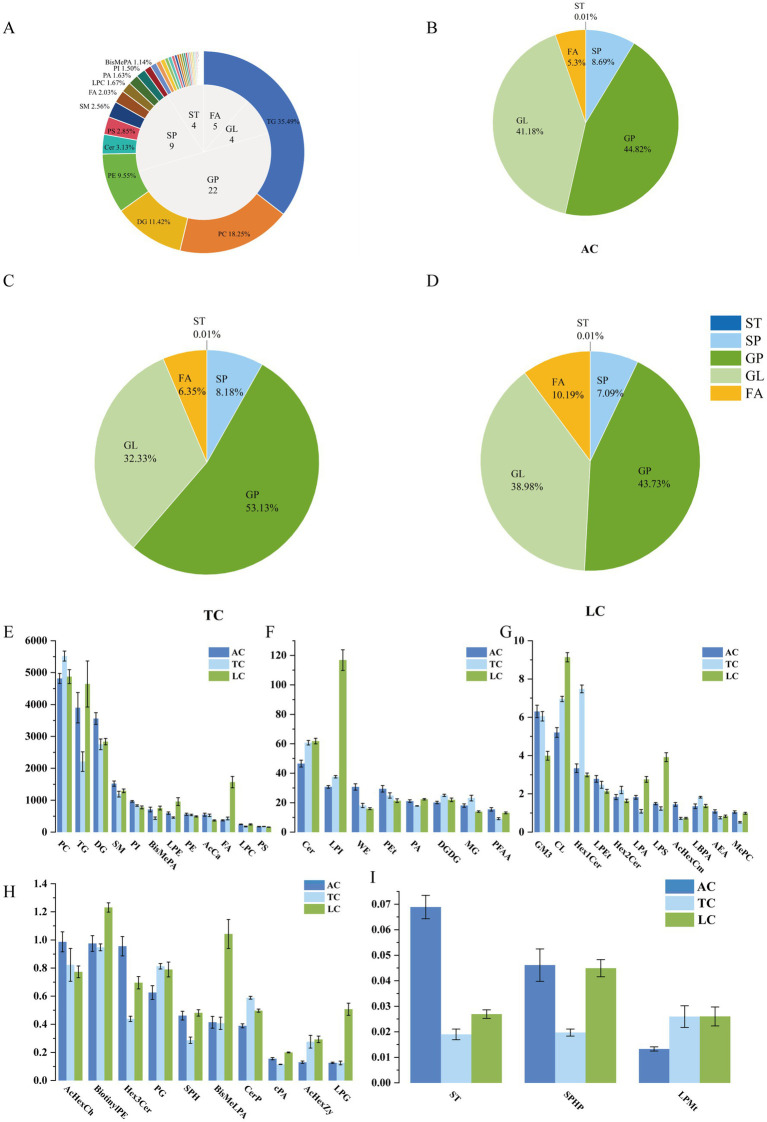
Count and proportion of subclasses within 5 lipid classes **(A)**. Relative signal distribution of lipid subclass classification **(B–D)**. Internal-standard-normalized semi-quantitative responses of lipid subclasses in AC, TC, and LC **(E–I)**.

This study identified 2,460 lipid compounds across positive and negative ion modes, classified into 44 subclasses. Glycerophospholipids (22 subclasses) were the most diverse and abundant, with Phosphatidylcholine (PC, 449, 18.25%), Phosphatidylethanolamine (PE, 235, 9.55%), and Phosphatidylserine (PS, 70, 2.85%) as the top compounds. Sphingolipids had 6 subclasses, mainly Ceramides (Cer, 77, 3.13%), Sphingomyelins (SM, 63, 2.56%), and Monohexosylceramides (Hex1Cer, 14, 0.57%). Fatty acids included 5 subclasses: Fatty Acids (FA, 50, 2.03%), Wax Esters (WE, 12, 0.49%), Acylcarnitines (AcCa, 7, 0.28%). Glycerolipids had 4 subclasses, mainly Triacylglycerols (TG, 873, 35.49%), Diacylglycerols (DG, 281, 11.42%), and Monoacylglycerols (MG, 10, 0.41%) (Figure 4A). The remaining subclasses were sterol lipids, each with 1 subclass and low abundance. Glycerophospholipids showed the highest subclass diversity. The detected lipid signals were mainly contributed by TG, PC, and DG within this analytical framework.

The main lipid classes in camel milk across three regions were GP, followed by GL. Within this semi-quantitative dataset, the highest relative signal intensities were observed for lipid subclasses within GP and GL: PC, TG, and DG (Figures 4B–D). Differences in relative signal intensities across regions were observed, with PC showing the highest relative response in all areas within this analytical framework (Figures 4E–I).

### 3.3 Multilevel statistical analysis

**Figure 5:**
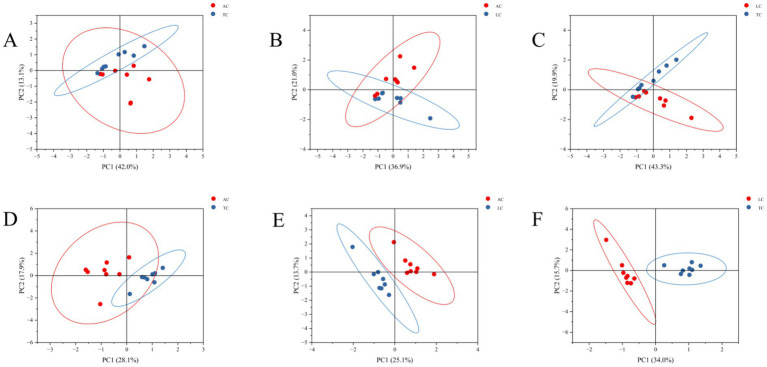
The PCA scores plot of AC vs. TC **(A)**, LC vs. AC **(B)** and LC vs. TC **(C)** in positive ion mode and plot of AC vs. TC **(D)**, LC vs. AC **(E)** and LC vs. TC **(F)** in negative ion mode.

**Figure 6:**
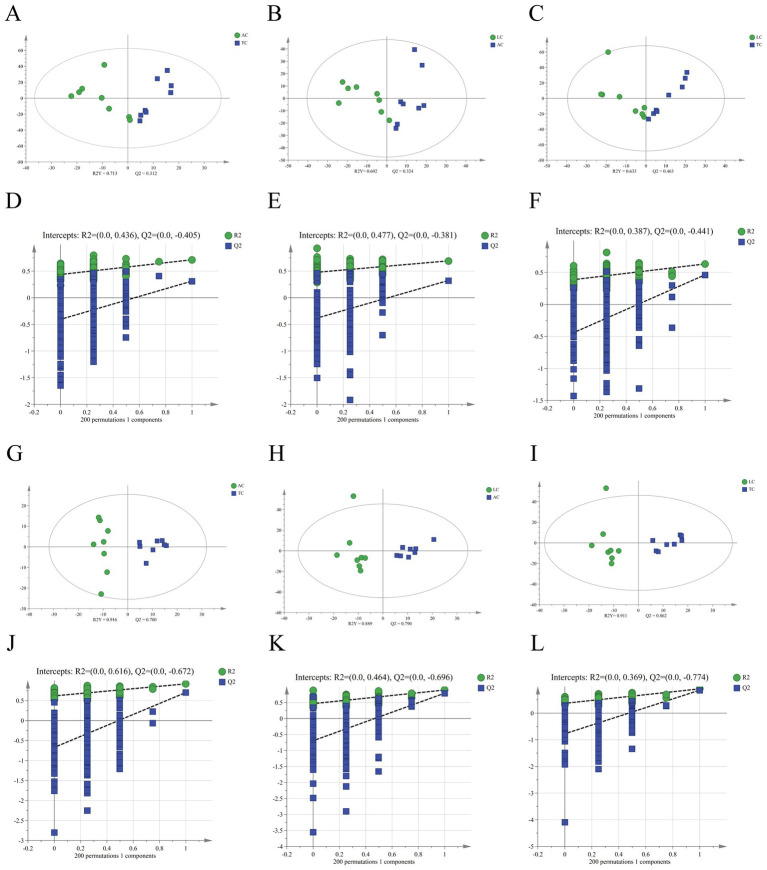
OPLS-DA score plot and OPLS-DA displacement test plot of AC vs. TC **(A,D)**, LC vs. AC **(B,E)** and LC vs. TC **(C,F)** in positive and AC vs. TC **(G,J)**, LC vs. AC **(H,K)** and LC vs. TC **(I,L)** in negative ion mode.

PCA and OPLS-DA revealed significant differences among camel milk samples from different regions in both positive and negative ion modes (Figures 5, 6), indicating distinct region-associated lipidomic profiles. These results support the presence of systematic lipid variation among AC, TC, and LC samples and provide a basis for subsequent screening of differential lipids.

### 3.4 Identification of significantly different lipids

**Figure 7:**
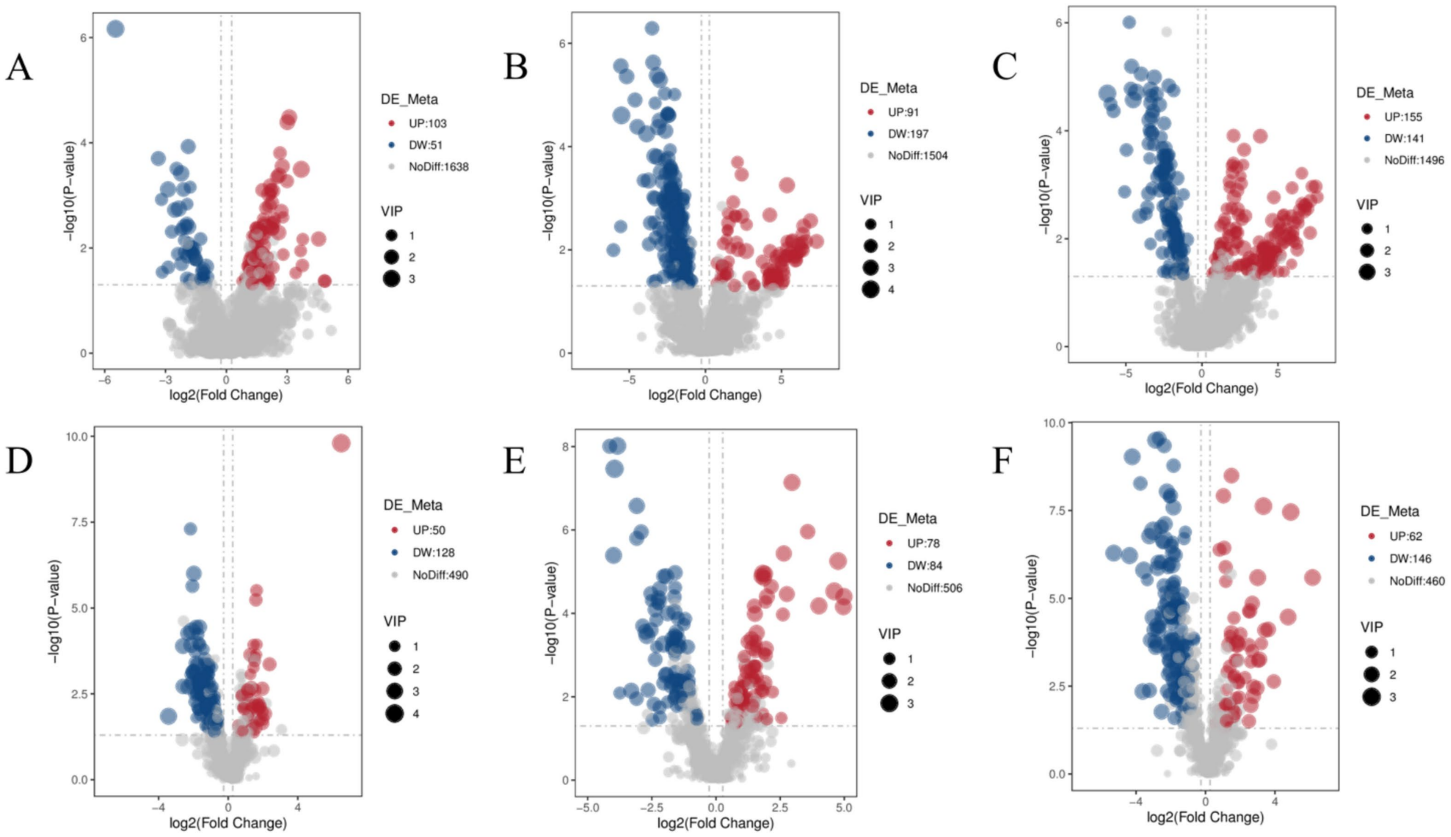
Volcano plot of differential lipid compounds of AC vs. TC **(A)**, LC vs. AC **(B)** and LC vs. TC **(C)** in positive ion mode and AC vs. TC **(D)**, LC vs. AC **(E)** and LC vs. TC **(F)** negative ion mode.

The differential lipid compounds in camel milk across three regions were screened using a multi-criteria statistical framework incorporating *p* < 0.05, FC ≥ 2 or ≤ 0.5, and VIP > 1.0. A total of 1,286 differentially expressed lipids were identified after screening. As shown in Figures 7A,D and Supplementary Table S2, 332 lipids were identified as significantly different between AC and TC, with 153 lipids showing significantly higher abundance in AC, including 44 PCs, 18 SMs, 13 PE, 8 DGs, 8 LPCs, and 27 other lipid classes. The remaining 179 lipids were significantly lower in AC compared to TC, including 43 PCs, 37 PE, 29 DGs, 18 Cers, 15 PSs, and 18 other lipid classes. 450 lipids exhibited significant differences between LC and AC, with 169 lipids showing significantly higher abundance in LC, including 58 TGs, 26 PCs, 15 PE, 14 DGs, 9 FAs, and 23 other lipid classes. The remaining 281 lipids were significantly lower in LC compared to AC, including 120 PCs, 54 PE, 30 DGs, 15 SMs, 14 PSs, and 21 other lipid classes (Figures 7B,E and Supplementary Table S3). There were 504 significantly different lipids between LC and TC, with 217 lipids showing significantly higher abundance in LC, including 89 TGs, 29 PCs, 12 PE, 11 DGs, 11 SMs, and 27 other lipid classes. The remaining 287 lipids were significantly lower in LC than in TC, including 101 PCs, 69 PE, 40 DGs, 19 PSs, 13 Cers, and 24 other lipid classes. (Figures 7C,F and Supplementary Table S4).

### 3.5 Screening of exploratory candidate lipids associated with regional separation

**Figure 8:**
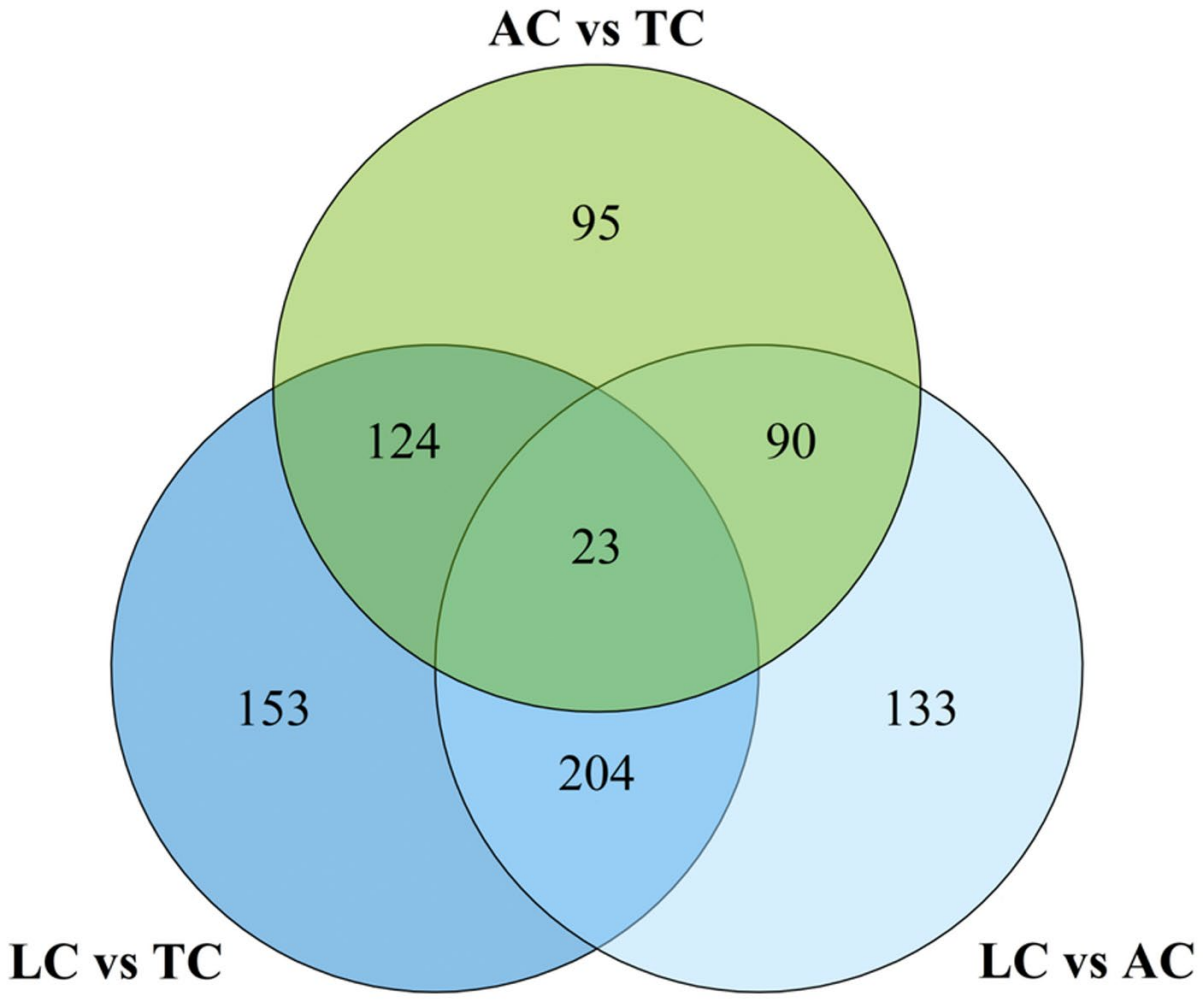
Venn diagram of significantly different lipids between three groups.

**Figure 9:**
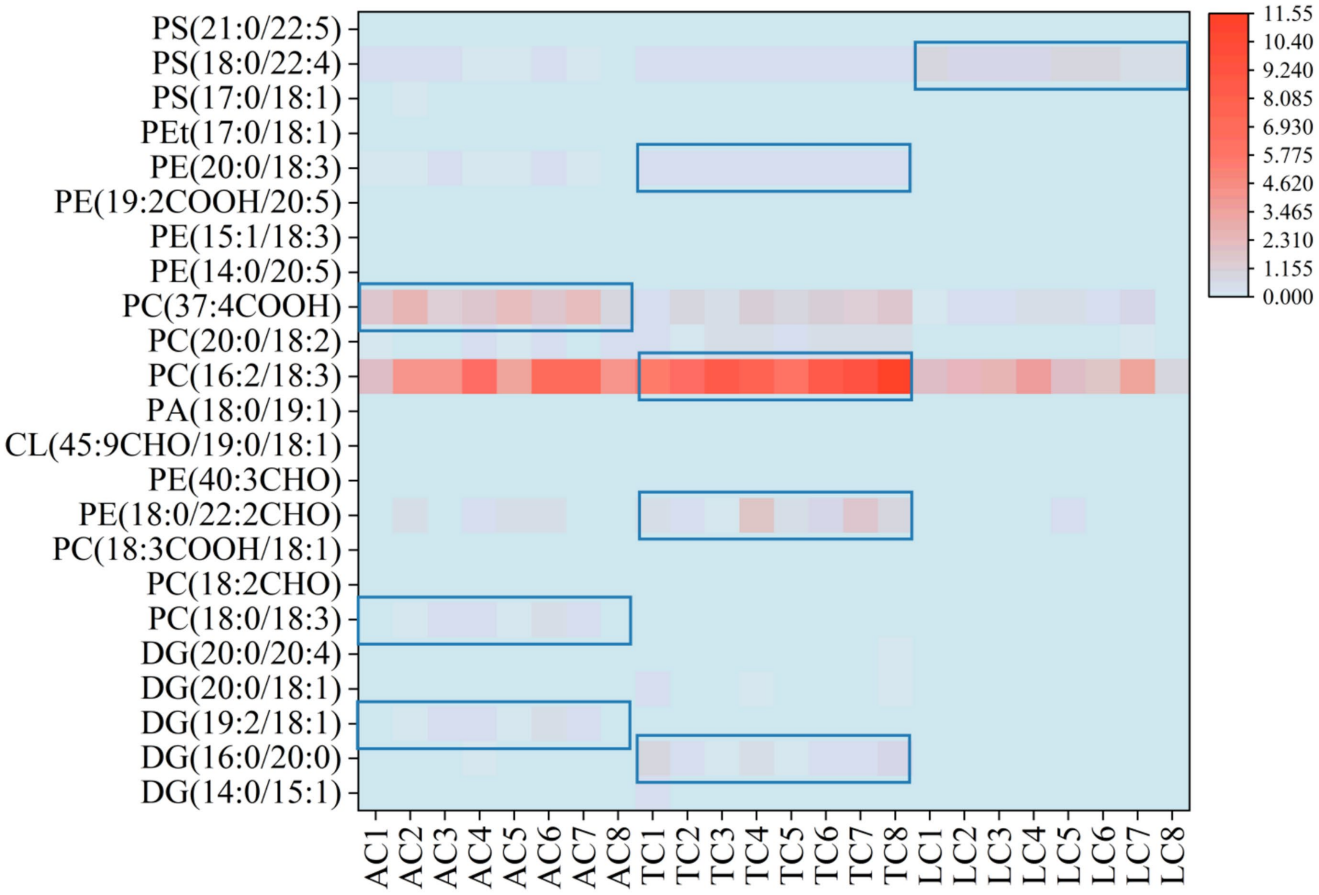
Heat map of significantly different lipids between AC, TC, and LC.

To identify candidate lipids for discriminating the geographical origins of camel milk, we performed a cross-comparative analysis of 1,286 significantly differential lipids across three regions. Venn diagram analysis revealed 23 overlapping differential lipid molecules (Figure 8; Supplementary Table S5). Following manual validation and selection, exploratory candidate lipids were identified. As shown in Figure 9, the levels of PC(37:4COOH), PC(18:0/18:3), and DG(19:2/18:1) in AC were higher than those in TC and LC. In TC, four lipid species had higher levels than in AC and LC: PC(16:2/18:3), PE(18:0/22:2CHO), PE(20:0/18:3), and DG(16:0/20:0). LC showed higher PS(18:0/22:4) content than AC and TC. In summary, eight lipid molecules showed region-associated patterns among AC, TC, and LC, and may represent exploratory candidate markers for future traceability studies. PE, PC, and DG were the main lipid subclasses among the exploratory candidate lipids in AC and TC. PS was the main lipid subclass among the exploratory candidate lipids in LC.

**Figure 10:**
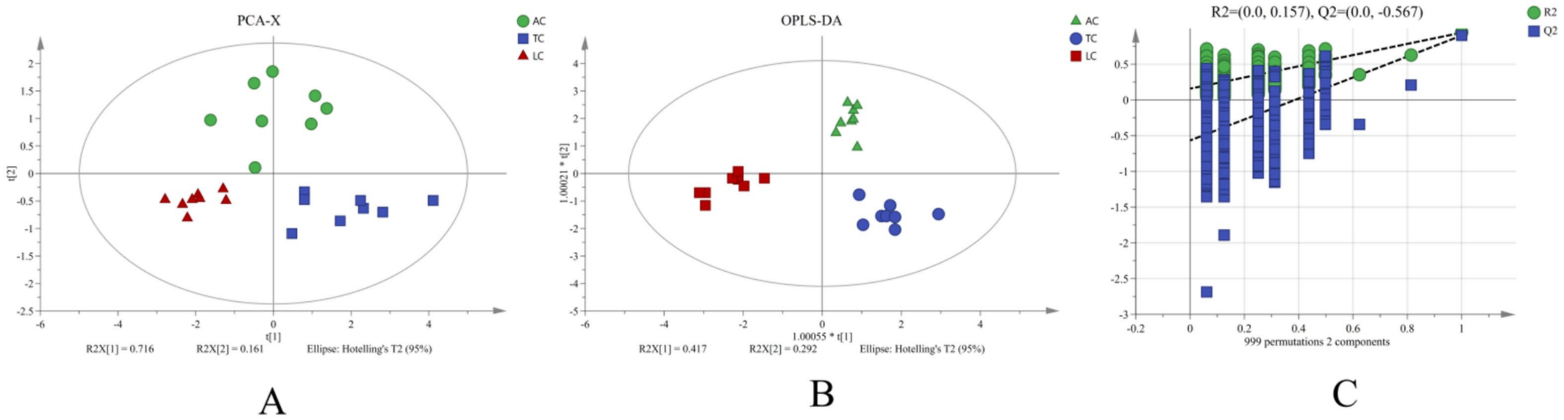
Score plots of PCA-X **(A)** and OPLS-DA **(B)** for candidate markers and the corresponding permutation test (999 permutations) **(C)**.

To assess whether these differential lipids carried regional discriminatory information, PCA was performed to classify the samples based on lipid molecular species. According to the PCA score plot, samples from the same category clustered together, and the three groups were clearly separated (Figure 10). To elucidate this differentiation, the first two principal components (PC1 and PC2) were extracted from the PCA. The PCA model explained 71.6% of the variance with PC1 and 16.1% with PC2, accounting for a cumulative total of 87.7%. Samples from different collection regions clustered distinctly and were clearly segregated into three groups corresponding to AC, TC, and LC. Based on the quantitative relative abundances of the candidate lipids identified above, an OPLS-DA classification model was established. Clear separation between the groups was achieved. The OPLS-DA model underwent permutation testing (999 permutations) to assess its validity. Permutation testing indicated limited predictive robustness of the model (*R*^2^ = 0.0157, *Q*^2^ = −0.567). These results indicate region-associated differences in the relative abundances of the eight lipid molecules—PC(37:4COOH), PC(18:0/18:3), DG(19:2/18:1), PC(16:2/18:3), PE(18:0/22:2CHO), PE(20:0/18:3), DG(16:0/20:0), and PS(18:0/22:4)—in camel milk from different geographical origins. Consequently, this eight-lipid panel may aid exploratory discrimination of camel milk sourced from AC, TC, and LC, but requires independent validation.

### 3.6 Identification of differential oxidized lipids

Notably, under the criteria of FC ≥ 1.5 or ≤ 0.667 and *p* < 0.05, we identified five distinct types of oxidized lipids derived from polyunsaturated fatty acids (PUFAs): arachidonic acid (AA), linoleic acid (LA), eicosapentaenoic acid (EPA), docosahexaenoic acid (DHA), and others. In the AC vs. TC comparison (Supplementary Table S6), 8 AA-derived, 3 DHA-derived, 1 EPA-derived, 3 LA-derived, and 1 other were upregulated, and 2 AA-derived and 1 LA-derived were downregulated. In the LC vs. AC comparison (Supplementary Table S7), 3 AA-derived, 1 DHA-derived, 1 EPA-derived, 6 LA-derived, and 1 Other were upregulated, and 4 AA-derived, 4 DHA-derived, and 5 EPA-derived were downregulated. In the LC vs. TC comparison (Supplementary Table S8), 7 AA-derived, 1 EPA-derived, 7 LA-derived, and 1 other were upregulated, and 5 AA-derived, 2 DHA-derived, and 4 EPA-derived were downregulated.

Regional forage differences may contribute to variation in unsaturated fatty acid inputs to camel milk (exceeding 50%), making them one of the most cost-effective sources of unsaturated fatty acids in animal diets (17). Because n-3/n-6 PUFA balance can influence downstream oxylipid metabolism, regional differences in PUFA-derived oxylipids may be biologically relevant (18). Notably, 15-HEPE, 9-HETE, and AA were detected across all three regions. 5-HEPE was downregulated in both the LC vs. AC and LC vs. TC comparisons, whereas 9-HETE was upregulated exclusively in the AC vs. TC and LC vs. TC comparisons. Previous studies have linked AA metabolism to hypoxia-related responses, but the relevance of this mechanism to the present milk dataset remains inferential (19). In the AC vs. TC regional comparison, the significant upregulation of AA-derived metabolites (e.g., 9-HETE, 8-HETrE) is consistent with a region-associated shift in oxylipid metabolism. Our findings suggest that ecological context is associated with variation in camel milk oxylipid profiles.

### 3.7 Metabolic pathway enrichment analysis

**Figure 11:**
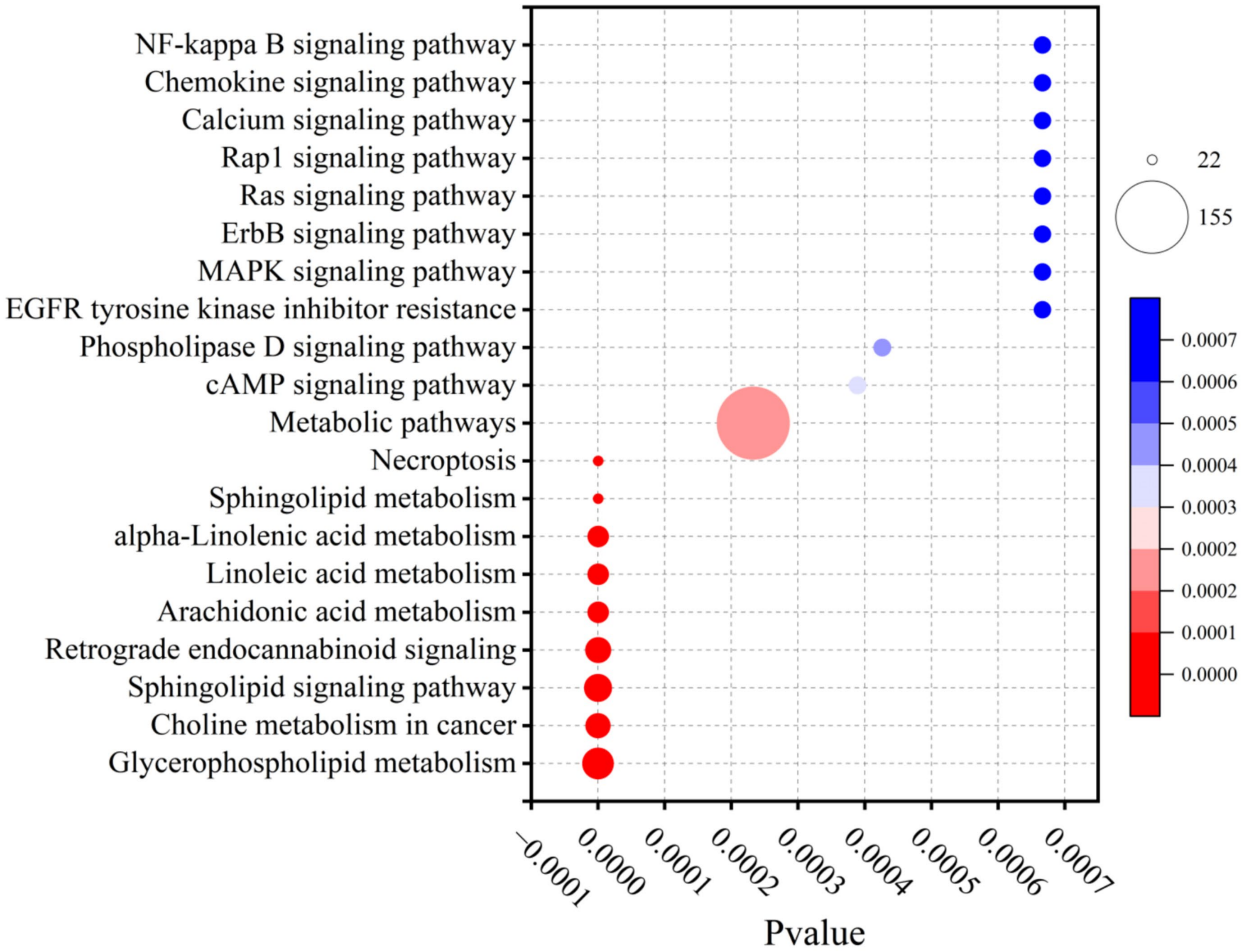
KEGG pathway analysis of camel milk differential lipid compounds.

To determine whether lipid metabolism varied by region, significantly altered lipids were mapped to the KEGG database. This identified 70 relevant metabolic pathways, of which 37 showed significant differences (*p* < 0.05). The top 20 most significantly enriched lipid metabolic pathways, ranked by *p*-value (*p* < 0.05), were visualized. The most significantly enriched pathways included glycerophospholipid metabolism, choline metabolism in cancer, sphingolipid signaling pathway, retrograde endocannabinoid signaling, arachidonic acid metabolism, linoleic acid metabolism, and alpha-linolenic acid metabolism. In the enrichment plot, these pathways appeared as darker, larger bubbles, indicating stronger associations (Figure 11). Notably, robust activity in oxidized lipid metabolism— specifically arachidonic acid, linoleic acid, and alpha-linolenic acid metabolism pathways— was observed. This finding aligns with the differentially altered lipid compounds identified in our earlier analysis.

## 4 Discussion

This study characterized region-associated lipid variation in camel milk collected from three ecologically distinct areas of Xinjiang by integrating untargeted lipidomics with targeted oxylipidomics. In addition to broad differences in phospholipids, glycerolipids, and sphingolipids, the study also captured regional variation in PUFA-derived oxidized lipids, thereby extending previous compositional studies of camel milk beyond conventional lipid profiling alone. Together, these findings indicate that the camel milk lipidome is sensitive to ecological context and may preserve descriptive biochemical signatures associated with regional production environments. Compared with previous regional compositional studies of camel milk, the present work extends the evidence base by jointly profiling untargeted lipids and targeted PUFA-derived oxylipids across three ecologically distinct regions.

PCs are vital components of the cell membrane bilayer. Their production requires various enzymes and metabolic routes, such as lysophosphatidylcholine acyltransferase (LPCAT) and choline-phosphate cytidylyltransferase (CTP) (20). Notably, TC exhibited higher PC content than the other regions, potentially associated with intensified drought stress (i.e., the lowest annual precipitation). Desert steppe vegetation may be associated with altered phosphatidylcholine-related metabolic patterns in camels (21), although the mechanistic relevance of this interpretation to mammary lipid secretion remains to be verified (22). Previous research demonstrated increased milk fat in goats under low-temperature conditions (−3 to 6 °C, 63% humidity, THI = 33–46) (23). The highest TG-related signals observed in LC may be due to differences in forage quality under local climatic conditions. The elevated TG/DG ratio in LC (4643.83/2837.34 ≈ 1.64) compared to TC (2209.98/2753.22 ≈ 0.80) and AC (3898.78/3560.01 ≈ 1.10) may indicate a shift in lipid storage–related patterns under local grazing conditions.

The regional differences in the abundance of these lipid categories (e.g., PCs, TGs, DGs) not only highlight the profound influence of the geographical environment on the lipid metabolic network of camel milk but also provide molecular evidence of region-associated lipid remodeling in camel milk. Differences in the grazed pasture may lead to variations in milk lipid composition (24). Research indicates that when dietary conjugated linoleic acid (CLA) supplements are provided, the levels of PC, LPE, and PE in bovine milk decrease, while the levels of PI and PS increase (25). This phenomenon underscores the potent regulatory capacity of dietary sources on key milk lipid constituents, particularly phospholipids with critical structural functions. A comparative analysis of AC versus TC and LC revealed GP (e.g., PC, PE) as the most significantly altered lipid category, followed by SP (e.g., SM) and GL (e.g., DG). Notably, AC had the lowest annual average temperature among the three regions, and camel diets were dominated by super-xerophytic shrubs and halophytic plants rich in unsaturated fatty acids (e.g., linoleic and linolenic acids) (26). These fatty acids serve as essential substrates for phospholipid synthesis (27). When incorporated into phospholipid molecules -- the fundamental scaffold of cell membranes—they substantially enhance bilayer fluidity and molecular flexibility. Under relatively low-temperature conditions (e.g., AC region), such unsaturated fatty acid-enriched phospholipids may help maintain membrane fluidity, thereby maintaining normal membrane permeability and functionality (e.g., substance transport, signal transduction) (28).

This may be related to regional forage types: AC and TC regions are desert and semi-desert areas dominated by drought-tolerant shrubs, with the lowest proportion of graminaceous grasses. Halophytes also constitute a certain proportion. Conversely, the LC grassland has the highest productivity, the highest average annual precipitation, the highest average annual temperature, and a significantly higher proportion of high-quality graminaceous grasses. PS is a common phospholipid species. It is predominantly located in the inner leaflet of the plasma membrane and is a major constituent of eukaryotic cell membranes. PS is involved in membrane function, serves as a cofactor for various enzymes, and is considered to play important roles in cellular excitability and signaling (29, 30). Its physiological relevance in camel milk remains unclear (31).

Although a systematic screening process was used to identify the eight exploratory candidate lipids, the selection included manual validation steps that could introduce subjective bias. While the OPLS-DA model showed clear separation between groups, the low *Q*^2^ value indicates limited predictive reliability and suggests a high risk of overfitting, likely due to the small sample size relative to the number of variables. Consequently, these candidate lipids should be viewed as preliminary, and their ability to discriminate needs to be confirmed through rigorous validation with targeted quantitative methods in larger, independent cohorts before they can be considered reliable markers for the geographical traceability of camel milk.

Camel milk exhibited high GP content (AC: 44.82%; TC: 53.13%; LC: 43.73%). GPs undergo continuous conversion, including enzymatic hydrolysis to liberate PUFAs. Under the influence of cyclooxygenases, lipoxygenases, and cytochrome P450 enzymes, these PUFAs are metabolized into various oxidized lipids. Among oxidized lipids, eicosanoids derived from AA are widely studied bioactive mediators. Their levels can vary with physiological and environmental context. These mediators have been implicated in redox and inflammatory regulation in multiple biological systems (32). Some previous studies have linked oxidized linoleic acid metabolites to antioxidant signaling pathways in other biological systems (33–35). However, the present study did not directly assess skin physiology or adaptive phenotypes, so such mechanisms remain speculative in the context of camel milk. Concurrently, dietary plant-derived *α*-linolenic acid may modulate fatty acid profiles and potentiate health benefits (36). Pharmacological studies indicate that α-linolenic acid exhibits anti-inflammatory, anti-obesity, and anti-cancer properties, combats metabolic syndrome and oxidative stress, and demonstrates neuroprotective effects and gut microbiota-modulating capabilities (37). Overall, the observed oxylipid differences are best interpreted as region-associated metabolic variation in camel milk.

Several limitations of this study should be acknowledged. First, the sample size per region was relatively small, which may affect the stability of the predictive classification models. Second, the untargeted lipidomic data were based on internal-standard-normalized semi-quantitative abundances rather than strict absolute quantification across all lipid subclasses, and the reported differences among lipid subclasses were generated within this analytical framework. Third, direct fatty acid profiling of camel milk and compositional analysis of regional forage were not performed, limiting further exploration of the biological basis underlying the observed lipid differences. Fourth, all samples were collected within a single season, and the temporal stability of the proposed candidate markers has yet to be determined. Despite these limitations, the present study provides a comprehensive lipidomic dataset for camel milk from Xinjiang and demonstrates that both structural lipids and PUFA-derived oxidized lipids vary across ecological regions. These findings offer molecular-level evidence for region-associated heterogeneity in camel milk and establish a basis for future studies on geographical traceability, targeted validation, and nutritional characterization.

## 5 Conclusion

This study used UHPLC–MS/MS-based untargeted lipidomics and targeted oxylipidomics to identify 44 lipid subclasses comprising 2,460 lipid molecules in camel milk across three distinct regions of Xinjiang, China. Screening revealed 1,286 significantly altered lipids and highlighted 8 exploratory candidate lipids associated with regional separation. KEGG pathway mapping of differentially abundant lipids identified 37 significantly regulated metabolic pathways. This work provides an initial foundation for future validation of lipid markers to discriminate camel milk by geographical origin within Xinjiang. Overall, this study offers a preliminary molecular characterization of region-associated lipid heterogeneity in camel milk, laying the groundwork for subsequent validation studies.

## Data Availability

The data presented in the study have been deposited in the Mendeley Data repository, DOI: 10.17632/zdmts8bnjn.2, dataset ID: zdmts8bnjn, version 2. The dataset is available at: https://data.mendeley.com/datasets/zdmts8bnjn/2.

## Footnotes

1
https://www.genome.jp/kegg/pathway.html

2
https://hmdb.ca/metabolites

3
http://www.lipidmaps.org/
